# EEG Beta functional connectivity decrease in the left amygdala correlates with the affective pain in fibromyalgia: A pilot study

**DOI:** 10.1371/journal.pone.0281986

**Published:** 2023-02-21

**Authors:** Soline Makowka, Lliure-Naima Mory, Michael Mouthon, Christian Mancini, Adrian G. Guggisberg, Joelle Nsimire Chabwine

**Affiliations:** 1 Faculty of Science and Medicine, Department of Neuroscience and Movement Science, Laboratory for Neurorehabilitation Science, Medicine Section, University of Fribourg, Fribourg, Switzerland; 2 Neurorehabilitation Division, Fribourg Hospital Meyriez/Murten, Fribourg, Switzerland; 3 Department of Clinical Neuroscience, Division of Neurorehabilitation, Geneva University Hospital, Geneva, Switzerland; University of Würzburg, GERMANY

## Abstract

Fibromyalgia (FM) is a major chronic pain disease with prominent affective disturbances, and pain-associated changes in neurotransmitters activity and in brain connectivity. However, correlates of affective pain dimension lack. The primary goal of this correlational cross-sectional case-control pilot study was to find electrophysiological correlates of the affective pain component in FM. We examined the resting-state EEG spectral power and imaginary coherence in the beta (β) band (supposedly indexing the GABAergic neurotransmission) in 16 female patients with FM and 11 age-adjusted female controls. FM patients displayed lower functional connectivity in the High β (Hβ, 20–30 Hz) sub-band than controls (*p* = 0.039) in the left basolateral complex of the amygdala (*p* = 0.039) within the left mesiotemporal area, in particular, in correlation with a higher affective pain component level (r = 0.50, *p* = 0.049). Patients showed higher Low β (Lβ, 13–20 Hz) relative power than controls in the left prefrontal cortex (*p* = 0.001), correlated with ongoing pain intensity (r = 0.54, *p* = 0.032). For the first time, GABA-related connectivity changes correlated with the affective pain component are shown in the amygdala, a region highly involved in the affective regulation of pain. The β power increase in the prefrontal cortex could be compensatory to pain-related GABAergic dysfunction.

## Introduction

Fibromyalgia (FM) is one of the most frequent chronic pain disease, reaching up to 4% of frequency in the general population, and affecting more significantly females than males [1]. The clinical constellation characterizing FM combines widespread chronic pain, fatigue, mood disorders, cognitive deficits and sleep disturbances. Despite active research on underlying mechanisms and etiologies, FM remains a poorly understood disease condition.

As in any other chronic pain syndrome, central sensitization plays an important role in FM [2], which could be reflected by altered brain dynamics in several areas involved in nociception observed in functional connectivity (FC) studies [3]. A recent resting-state fMRI study found transient functional connectivity changes related to ongoing pain intensity, driven by central sensitization in brain areas responsible for pain regulation in FM patients [4]. However, the study did not discriminate between different dimensions of pain, which could have contributed to further understand mechanisms subtending observed connectivity modifications. Knowing the prominent impact of emotional disturbances in FM, we made the hypothesis that the affective pain component would be related to connectivity changes in emotional pain-regulating brain regions [4].

Chronic pain induces significant modifications in neurotransmitter pathways, the most remarkable among them being an over-excitatory state owing to a decrease in the inhibitory input [5] and/or excessive excitatory neurotransmission [6]. Accordingly, substantial decrease in brain GABAergic signaling is reported in chronic pain patients [7], brain inhibition being mainly driven by GABAergic interneurons [8]. Furthermore, we recently showed reduction in EEG markers of the GABAergic neurotransmission, namely beta (β) oscillations, in chronic neuropathic pain [9]. Thus, we further assumed that expected power and FC changes in FM would be measurable through decrease in the EEG β oscillatory band.

This pilot study is part of a larger project investigating β EEG oscillations considered to be indicators of the GABAergic neurotransmission in chronic pain clinical models as a contribution to the mechanistic approach of pain characterization and therapy. In this particular investigation, the aim was to assess EEG FC changes in the β frequency domain occurring in FM patients and relate observed modifications to the affective component of pain.

## Materials and methods

### Study design

This correlational, cross-sectional case-control pilot study included FM patients and age- and sex-adjusted healthy participants (2018–2020). Approval by the Ethical Committee of Vaud (CER-VD) was obtained under the number PB_2016–00739 (initial number 331/15). Each participant signed an informed consent form prior to any data collection and received a financial compensation thereafter.

Patients were recruited mainly through neurologists, rheumatologists and pain specialists from Fribourg Hospital, and through Swiss FM associations using web-based and flyer advertisements, while advertisements for controls targeted middle-aged adult hobby associations. Participants (cases and controls) were adult (≥18 y) females [1] and right-handed [9]. The diagnosis of FM had to be made by the specialists and meet internationally admitted criteria (see below). Exclusion criteria consisted in: existence of central nervous system lesion or disease (including epilepsy and parasomnia), significant cognitive impairment, coexistence of any other type of pain (patients) or any pain (controls), and surgery involving any nervous system structure less than six months before inclusion [9].

### Data collection

Each participant was interviewed following a standardized questionnaire (age, sex, marital status, profession and education, treatments, relevant medical history) and underwent a brief neurological examination in order to exclude abnormalities potentially related to a central nervous system lesion or disease.

Three additional specific questionnaires (the FM Rapid Screening Tool (FiRST) [10], the Symptoms Severity Score (SSS) and the Widespread Pain Index (WPI) of the 2010 American College of Rheumatology criteria (ACR 2010) were used to confirm FM (FiRST > 5, SSS ≥ 7 and WPI ≥ 5 or SSS ≥ 9 and WPI 3–6 [11]).

Pain evaluation (FM patients) included pain intensity using the Visual Analogue Scale (VAS) on the day of evaluation (VAS_d_) and on average over the week before [12], considering VAS_d_≥3 as significant [9]. The VAS shows good statistical qualities for evaluation of chronic pain patients [13–15], and is frequently used in FM studies [16,17]. The Short-form 2 McGill Pain Questionnaire allowed differentiation between the sensory (SF-MPQ-2_sensory_) and the affective (SF-MPQ-2_affective_) components of pain (both scores re-scaled /10) [18]. The SF-MPQ-2 has been developed to be used in chronic pain populations and has excellent reliability and validity [18]. It has also been often used in FM studies [17,19–21]. The Hospital Anxiety and Depression Scale (HADS) determined existence of anxiety and depression [22], whereas the Insomnia Severity Index (ISI) was compiled for insomnia assessment [23]. HADS is a reliable instrument for screening clinically significant anxiety and depression as well as their severity [22,24], frequently used in FM studies [25–27]. Finally, ISI is a reliable and valid instrument to quantify insomnia severity [23,28]. It has already been used in chronic pain population [29,30], including in FM patients [31–33].

The EEG data were recorded using a high density 64-channel EEG recording system (BIOSEMI ActiveTwo, Amsterdam, Netherlands) at a sampling rate of 1024 Hz. All EEG recordings were obtained before noon [9] in a quiet dark room shielded by a Faraday cage. Participants were requested to sit down with eyes closed, minimizing eye blinks and body movements. The recording lasted 24 minutes. To avoid sleepiness due to the length of the recording, participants were maintained seated, and acoustic sounds were used two times during the recording.

#### EEG data

Raw EEG data were down-sampled to 512 Hz, and band-pass filtered between 0.5 and 40 Hz. Bad EEG channels were excluded by visual inspection using Cartool software for data visualization, while careful manual artifact-rejection was performed to exclude eye movements and blinks, body movements and electrode drifts. Only the first five minutes of artifact-free data of the recording was retained for the analysis.

Preprocessed data were referenced to the Cz electrode and segmented into non-overlapping 1-second epochs. Analyses were performed in MATLAB (The MathWorks), using the toolbox NUTMEG [34,35]. The lead-potential was computed using a boundary element head model [36,37], with the Helsinki BEM library [34] and the NUTEEG plugin of NUTMEG. The head model was based on the Montreal Neurological Institute template brain, and solution points were defined in the gray matter with 10 mm grid spacing.

EEG epochs were Hanning-windowed, Fourier transformed, and projected to gray matter voxels, using an adaptive filter (scalar minimum variance beamformer) [38] and the δ (0.5 to 3.5 Hz), θ (3.5 to 7.5 Hz), α (7.5 to 12.5 Hz), Low β (Lβ, 13–20 Hz) and High β (Hβ, 20–30 Hz) frequency bands were defined. The β band (13–30 Hz) [39], supposedly indicating brain GABAergic activity, was divided into Lβ and Hβ sub-bands following our previous observations [9,40,41], whereas the δ band was considered as a control frequency.

The absolute source spectral power was computed as the absolute squared signal amplitude, whereas the relative power was obtained by normalizing the power in each band to the mean power of all bands and dividing by their standard deviation; thus obtaining z-scores.

FC was assessed in source space (i.e. after source localization) as the statistical dependency between reconstructed activities at the different solution points. Analysis of FC was conducted as described previously [34,42]. We used the absolute imaginary component of coherence as index of FC and calculated the weighted node degree (WND) for each solution point as the sum of its coherence with all other cortical solution points [43]. In order to minimize EEG signal-to-noise ratio influence on FC, we normalized WND values using z-scores by subtracting the mean WND value of all voxels of the subject from the imaginary component of coherence values at each voxel and by dividing by the standard deviation over all voxels [44,45].

#### Statistical analysis

Statistical non-parametric mapping was used to compare patients to controls at all solution points of the cortex. Correction for multiple testing was obtained by defining a cluster-size threshold based on the cluster size distribution obtained after random reversions of original data [46]. This voxel-wise analysis revealed the topography of contrasts, which was complemented by anatomical region of interest (ROI) defined with the Julich atlas [47], and the later thereafter compared between patients and controls using an unpaired *t*-test.

Associations between EEG and clinical data were analyzed with Spearman correlation test (more robust to detect outliers than Pearson correlation). Data are all presented as mean (SD) and the level of significance admitted at *p*<0.05 (95% confidence interval).

Voxel-wise statistics were performed with the toolbox NUTMEG, the remaining analyses with the Statistics toolbox of MATLAB (The MathWorks) [34,35].

## Results

### General data

**Fig 1:**
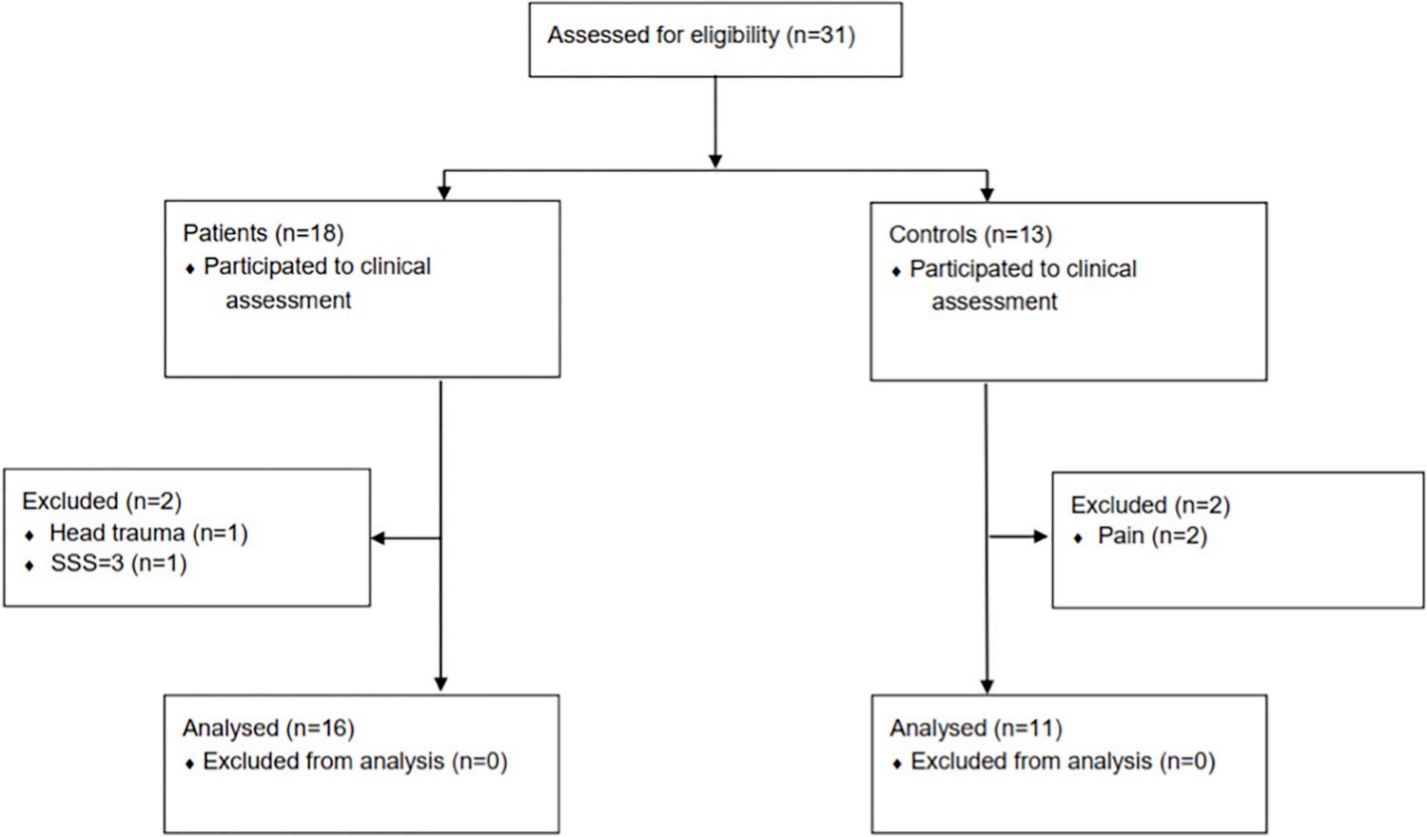
Participant selection procedure. In total, 31 participants were screened (18 fibromyalgia patients and 13 controls). Two patients were excluded because one had head traumatism and the other a symptoms severity scale (SSS) of 3 (the lowest SSS limit for fibromyalgia diagnosis was 7). Two controls were excluded as they complained of pain the day they were assessed. Finally, 16 patients and 11 controls were included in the study.

In total, 16 patients and 11 controls (51.8(8.5) and 54.2(4.6) years) were included for the analysis, as shown in the selection flowchart (Fig 1). FM patients complained of the typical widespread pain at moderate intensity the day of evaluation (VAS_d_ 4.75(2.84)) and during the last week before assessment (6.38(2.11)). The SF-MPQ-2_affective_ and SF-MPQ-2_sensory_ scores were similar (5.88(2.87) and 5.29(2.33), respectively). Patients reported moderate insomnia (ISI 17.75(5.27)), anxiety (HADS anxiety sub-score 10.50(4.12)) and depression (HADS depression sub-score 10.94(3.07)), while controls had normal scores (respective *p* <0.001, <0.005 and <0.001). Information regarding the patient’s medications are detailed in Table 1.

**Table 1:** FM patient’s medications.

TREATMENT	Type of treatment	n/16
	NSAID*	7
	Antidepressants	7
	Physical and alternative	5
	Antimigrainous	4
	Benzodiazepines	3
	Opiates	2
	Other drugs	5
	None	3

*NSAID: Nonsteroidal Anti-Inflammatory Drugs.

### Spectral power analysis

**Fig 2:**
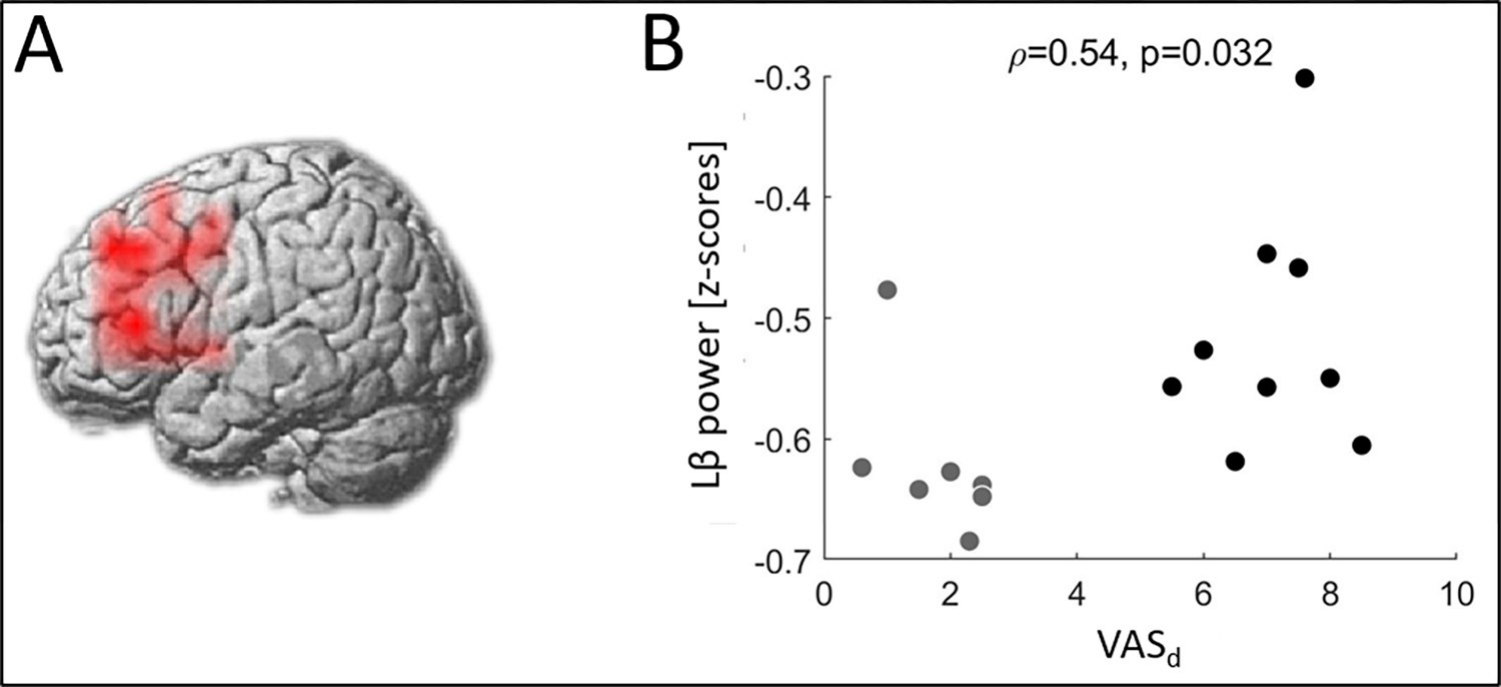
Low β (Lβ) relative power in fibromyalgia (FM) patients vs. controls. A voxel-wise analysis of the entire cortex (see methods for details) revealed a significant cluster of increased Lβ band (13–20 Hz) relative power in FM patients compared to controls (respective mean(SD)) of -0.56(0.10) and -0.69(0.06)) in the prefrontal cortex (red color, *p*<0.05, cluster corrected) **(A)**. This increase correlated with the ongoing pain intensity (VAS_d_) **(B)**. Grey dots correspond to patients with VAS_d_<3 whereas black dots are related to patients with significant pain (VAS_d_≥3).

Absolute source power values displayed no difference between FM patients and controls, while a significant cluster of higher Lβ relative power was observed in FM patients in the left prefrontal cortex (PFC) (Fig 2A), exclusively correlated with the VAS_d_ (ρ = 0.54, *p* = 0.032) (Fig 2B). FM patients with VAS_d_≥3 displayed higher relative Lβ power than those with VAS_d_<3 (*p* = 0.028). Neither difference nor correlation were seen in the δ band.

### Functional connectivity

**Fig 3:**
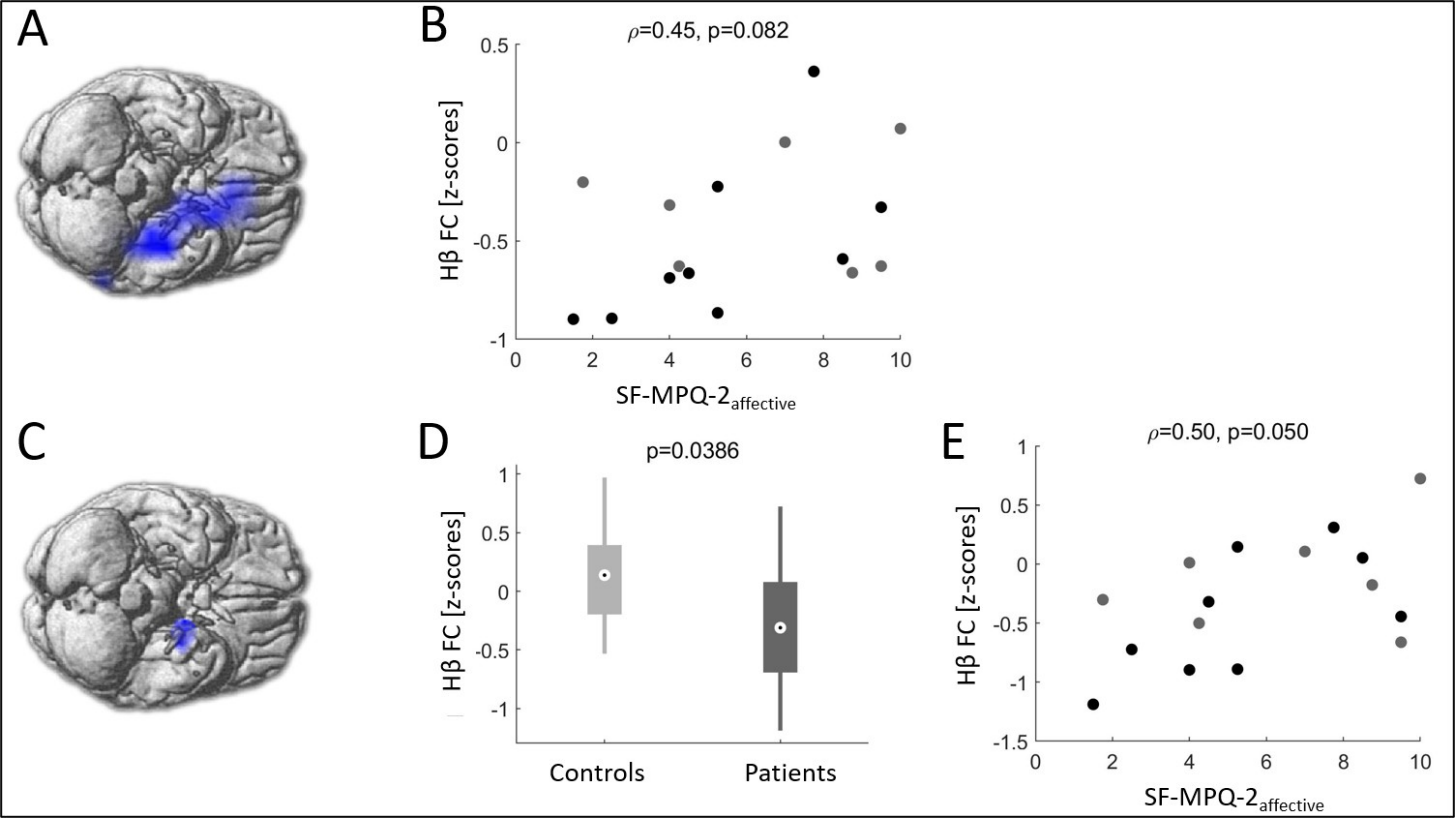
High β (Hβ) functional connectivity (FC) in fibromyalgia (FM) patients vs. controls. The voxel-wise analysis (see methods for details) revealed decreased Hβ (20–30 Hz) FC in FM patients in comparison to controls in the left mesiotemporal area (blue color, *p*<0.05, cluster corrected) **(A)**. A correlation was observed between Hβ FC of FM patients and the affective component of pain (SF-MPQ-2_affective_ scaled to 10), with a trend to significance **(B)**. Within the mesiotemporal area, the basolateral amygdala (BLA) showed a significant FC decrease (blue color, *p*<0.05) **(C)**. Corresponding quantitative values were respectively (mean(SD)) -0.30(0.51) in patients (dark grey color and 0.11(0.44) in controls (light grey) **(D)**. In addition, FC was significantly correlated with SF-MPQ-2_affective_ **(E)**. Grey dots correspond to patients with VAS_d_<3 whereas black dots are related to patients with significant pain (VAS_d_≥3).

A significant decrease in Hβ FC was noticed in FM patients compared to controls in the left mesiotemporal area (Fig 3A), with a trend to correlation with the SF-MPQ-2_affective_ (ρ = 0.45, *p* = 0.082) (Fig 3B). Within the left mesiotemporal area, the basolateral amygdala (BLA) displayed a significant decrease in Hβ FC (*p* = 0.039; Fig 3C and 3D), and a significant correlation with the SF-MPQ-2_affective_ (ρ = 0.50, *p* = 0.0495), with no impact of ongoing pain. No FC difference appeared in Lβ and δ frequencies, and the Hβ FC did not correlate with the other clinical scores.

## Discussion and conclusion

The most remarkable result of this study is the decrease of FM patients’ Hβ FC in the BLA and its selective correlation with the affective pain component. For the first time, a clear anatomo-clinical basis for the affective dysfunction in FM can be demonstrated, involving a brain area eloquent for the expression and control of emotions, as well as the modulation of the affective dimension of pain [48]. Furthermore, the BLA receiving all sensory (including the nociceptive) inputs, is believed to add an emotional valence to the latter before conveying them onto the central nucleus of the amygdala, its main output region [49]. Differences and correlations to pain descriptors confined to the β oscillatory domain indicate a GABAergic dysfunction in FM [50]. Moreover, in accordance with previous hypotheses [9], these data further suggest Hβ modifications as an indicator of pain-related affective dysfunction involving the BLA (rich of GABAergic interneurons [51]) in FM.

The ability of surface EEG to probe amygdala activity is controversial, given the inherently low signal to noise ratio in deep brain structures. Recent data suggest that high density EEG can reliably sense subcortical electrophysiological activity (including in the amygdala) [52–57]. However, validation with intra-cortical recordings have only been obtained in studies using higher-density EEG montages [53,58], while we used only 64 electrodes. One should also be cautious extrapolating results from patients with coma [57] and epilepsy [56] to patients with fibromyalgia. Nevertheless, our data overall suggest a GABA-mediated functional disturbance of brain activity (pointing to the amygdala) related to the affective dimension of pain in FM. Alterations in brain function have previously been observed in FM studies assessing FC by other methods such as magnetoencephalography [59], or investigating different brain networks such as the default mode network or the salience network [3]. However, none of these studies associated observed electrophysiological changes to specific neurotransmitter pathways or to measures of the affective pain component. Nonetheless, they all add up to the evidence for existence of objective brain dysfunction in FM.

Although Lβ power correlated with ongoing pain intensity as previously observed [9], to our surprise, there was now an increase and a positive correlation, contrary to the previously noticed decrease and negative correlation. Additionally, Lβ power maxima were previously observed in the posterior left-brain area (possibly corresponding to the somatosensory cortical areas) in contrast to the present Lβ power increase in the left PFC. Brain networks involved in pain chronification processes are similar to those implicated in executive functions (engaged in adaptive brain mechanisms) primarily controlled by the PFC [60], the latter being also implicated in pain regulation through abundant connections with the somatosensory system [61]. Considering the decrease in GABA-dependent inhibition occurring in chronic pain, we hypothesize that the increase in Lβ power possibly indicates a compensatory mechanism counteracting chronic pain-related GABAergic dysfunction. Interestingly, the PFC (in particular the dorsolateral PFC) constitutes a primary target for non-invasive brain therapies, such as the transcranial magnetic stimulation (TMS), including in FM [62]. Furthermore, TMS has been reported to have analgesic effects through GABAergic restauration [7]. Thus, the hypothesized “natural” GABA-related compensatory mechanisms would have potential analgesic effect, with possible reinforcement by therapeutic measures. Existing connections between the PFC and the amygdala [63] may finally provide an anatomic link between the identified site of brain dysfunction and the assumed compensatory region.

Previous investigations in FM reported both decrease [50] and increase [64] in GABAergic markers. In this study, Hβ decrease and Lβ increase were observed in different brain areas, associated to different pain descriptors, and differently interpreted (i.e. pathological decrease and compensatory increase), possibly reconciling these apparently conflicting results.

All depicted modifications in EEG markers occurred in left-sided brain areas in right-handed individuals, similar to previous observations in neuropathic pain and healthy populations [9], thereby supporting the concept of lateralization of chronic-pain-related brain modifications.

The specificity of the link between β EEG oscillations and pain can be questioned if we consider on one side, that GABAergic circuits also contribute to other oscillatory bands [7,65]. The current knowledge gives however, good indication for a link between fast EEG oscillations (namely β waves) and GABA concentrations in the brain [66,67]. Moreover, fast EEG oscillations are mainly driven by brain inhibitory interneurons, which are mostly GABAergic [67]. On the other side, we found an association between β waves and pain clinical descriptors, while at the same time, in the literature, β oscillations are linked with attention [68] or communication functions in verbal or non-verbal modalities [69]. Instead of seeing these findings as a contradiction with our results, we rather consider that β oscillations would indicate multifaceted aspects (including possibly the cognitive dimension) of pain. However, more investigations are warranted, to further enlighten this link. Additionally, due to the small number of participants in our research, further confirmation is necessary in larger studies. In addition, the interpretation frame of obtained results regarding the direction of electrophysiological modifications, their localization (i.e. decrease in the amygdala and increase in the PFC), as well as their implications in the mechanistic approach of pain in FM would need further investigation.

In conclusion, this study investigating EEG-measured GABAergic signaling modifications associated with the affective component of pain in FM patients, showed a Hβ FC decrease in the BLA correlated with the affective pain dimension, but an increase in PFC Lβ power associated with ongoing pain intensity. While the FC decrease was interpreted as part of the pathological process, the power increase was assumed to be compensatory, with potential therapeutic application. All disclosed modifications were left-sided, adding up to the emerging concept of left-lateralized changes in (GABAergic) pain-related brain pathways. Finally, given the small sample size and need for further methodological validation, larger and more accurate studies are needed to confirm these preliminary observations.

## References

[1] Wolfe F, Walitt B, Perrot S, Rasker JJ, Häuser W. Fibromyalgia diagnosis and biased assessment: Sex, prevalence and bias. Sommer C, editor. PLoS One. 2018;13: e0203755. doi: 10.1371/journal.pone.0203755 30212526 PMC6136749

[2] Cagnie B, Coppieters I, Denecker S, Six J, Danneels L, Meeus M. Central sensitization in fibromyalgia? A systematic review on structural and functional brain MRI. Seminars in Arthritis and Rheumatism. W.B. Saunders; 2014. pp. 68–75. doi: 10.1016/j.semarthrit.2014.01.001 24508406

[3] Vanneste S, Ost J, Van Havenbergh T, De Ridder D. Resting state electrical brain activity and connectivity in fibromyalgia. PLoS One. 2017;12. doi: 10.1371/journal.pone.0178516 28650974 PMC5484465

[4] Čeko M, Frangos E, Gracely J, Richards E, Wang B, Bushnell MC. Dependence of changes of the default mode network in fibromyalgia patients on current clinical pain. Neuroimage. 2020; 116877. doi: 10.1016/j.neuroimage.2020.116877 32344063 PMC8855626

[5] Henderson LA, Peck CC, Petersen ET, Rae CD, Youssef AM, Reeves JM, et al. Chronic pain: Lost inhibition? J Neurosci. 2013;33: 1754–1782. doi: 10.1523/JNEUROSCI.0174-13.2013 23616562 PMC6619566

[6] Parker RS, Lewis GN, Rice DA, Mcnair PJ. Is Motor Cortical Excitability Altered in People with Chronic Pain? A Systematic Review and Meta-Analysis. Brain Stimulation. Elsevier Inc.; 2016. pp. 488–500. doi: 10.1016/j.brs.2016.03.020 27133804

[7] Barr MS, Farzan F, Davis KD, Fitzgerald PB, Daskalakis ZJ. Measuring GAB aergic inhibitory activity with TMS-EEG and its potential clinical application for chronic pain. Journal of Neuroimmune Pharmacology. Springer; 2013. pp. 535–546. doi: 10.1007/s11481-012-9383-y 22744222

[8] Jones EG. Gabaergic neurons and their role in cortical plasticity in primates. Cereb Cortex. 1993;3: 361–372. doi: 10.1093/cercor/3.5.361-a 8260806

[9] Teixeira M, Mancini C, Wicht CA, Maestretti G, Kuntzer T, Cazzoli D, et al. Beta Electroencephalographic Oscillation Is a Potential GABAergic Biomarker of Chronic Peripheral Neuropathic Pain. Front Neurosci. 2021;15: 108. doi: 10.3389/fnins.2021.594536 33716642 PMC7952534

[10] Perrot S, Bouhassira D, Fermanian J. Development and validation of the Fibromyalgia Rapid Screening Tool (FiRST). Pain. 2010;150: 250–256. doi: 10.1016/j.pain.2010.03.034 20488620

[11] Wolfe F. New American College of Rheumatology Criteria for Fibromyalgia: A Twenty-Year Journey. Arthritis Care Res (Hoboken). 2010;62: 583–584. doi: 10.1002/acr.20156 20461781

[12] Collins SL, Moore RA, McQuay HJ. The visual analogue pain intensity scale: What is moderate pain in millimetres? Pain. 1997;72: 95–97. doi: 10.1016/s0304-3959(97)00005-5 9272792

[13] Price DD, Mcgrath PA, Rafii A, Buckingham B. The Validation of Visual Analogue Scales as Ratio Scale Measures for Chronic and Experimental Pain. Pain. 1983. 10.1016/0304-3959(83)90126-4 6226917

[14] Hawker GA, Mian S, Kendzerska T, French M. Measures of adult pain: Visual Analog Scale for Pain (VAS Pain), Numeric Rating Scale for Pain (NRS Pain), McGill Pain Questionnaire (MPQ), Short-Form McGill Pain Questionnaire (SF-MPQ), Chronic Pain Grade Scale (CPGS), Short Form-36 Bodily Pain Scale (SF-36 BPS), and Measure of Intermittent and Constant Osteoarthritis Pain (ICOAP). Arthritis Care Res. 2011;63. doi: 10.1002/acr.20543 22588748

[15] Hjermstad MJ, Fayers PM, Haugen DF, Caraceni A, Hanks GW, Loge JH, et al. Studies comparing numerical rating scales, verbal rating scales, and visual analogue scales for assessment of pain intensity in adults: A systematic literature review. Journal of Pain and Symptom Management. Elsevier; 2011. pp. 1073–1093. doi: 10.1016/j.jpainsymman.2010.08.016 21621130

[16] Cheatham SW, Kolber MJ, Mokha M, Hanney WJ. Concurrent validity of pain scales in individuals with myofascial pain and fibromyalgia. J Bodyw Mov Ther. 2018;22: 355–360. doi: 10.1016/j.jbmt.2017.04.009 29861234

[17] da Cunha Ribeiro RP, Franco TC, Pinto AJ, Pontes Filho MAG, Domiciano DS, de Sá Pinto AL, et al. Prescribed Versus Preferred Intensity Resistance Exercise in Fibromyalgia Pain. Front Physiol. 2018;9: 1097. doi: 10.3389/fphys.2018.01097 30158876 PMC6104489

[18] Dworkin RH, Turk DC, Revicki DA, Harding G, Coyne KS, Peirce-Sandner S, et al. Development and initial validation of an expanded and revised version of the Short-form McGill Pain Questionnaire (SF-MPQ-2). Pain. 2009;144: 35–42. doi: 10.1016/j.pain.2009.02.007 19356853

[19] Sánchez AI, Martínez MP, Miró E, Medina A. Predictors of the Pain Perception and Self-Efficacy for Pain Control in Patients with Fibromyalgia. Span J Psychol. 2011;14: 366–373. doi: 10.5209/rev_sjop.2011.v14.n1.33 21568193

[20] Geisser ME, Gracely RH, Giesecke T, Petzke FW, Williams DA, Clauw DJ. The association between experimental and clinical pain measures among persons with fibromyalgia and chronic fatigue syndrome. Eur J Pain. 2007;11: 202–207. doi: 10.1016/j.ejpain.2006.02.001 16546424

[21] Harris RE, Gracely RH, McLean SA, Williams DA, Giesecke T, Petzke F, et al. Comparison of Clinical and Evoked Pain Measures in Fibromyalgia. J Pain. 2006;7: 521–527. doi: 10.1016/j.jpain.2006.01.455 16814691

[22] Zigmond AS, Snaith RP. The Hospital Anxiety and Depression Scale. Acta Psychiatr Scand. 1983;67: 361–370. doi: 10.1111/j.1600-0447.1983.tb09716.x 6880820

[23] Morin CM, Belleville G, Bélanger L, Ivers H. The insomnia severity index: Psychometric indicators to detect insomnia cases and evaluate treatment response. Sleep. 2011;34: 601–608. doi: 10.1093/sleep/34.5.601 21532953 PMC3079939

[24] Bjelland I, Dahl AA, Haug TT, Neckelmann D. The validity of the Hospital Anxiety and Depression Scale: An updated literature review. J Psychosom Res. 2002;52: 69–77. doi: 10.1016/S0022-3999(01)00296-3 11832252

[25] Nam S, Tin D, Bain L, Thorne JC, Ginsburg L. Clinical utility of the Hospital Anxiety and Depression Scale (HADS) for an Outpatient Fibromyalgia Education Program. Clin Rheumatol. 2014;33: 685–692. doi: 10.1007/s10067-013-2377-1 23995734

[26] Vallejo MA, Rivera J, Esteve-Vives J, Rodríguez-Muñoz MF. Uso del cuestionario Hospital Anxiety and Depression Scale (HADS) para evaluar la ansiedad y la depresión en pacientes con fibromialgia. Rev Psiquiatr Salud Ment. 2012;5: 107–114. doi: 10.1016/j.rpsm.2012.01.003 22854581

[27] Marchi L, Marzetti F, Orrù G, Lemmetti S, Miccoli M, Ciacchini R, et al. Alexithymia and psychological distress in patients with fibromyalgia and rheumatic disease. Front Psychol. 2019;10: 1735. doi: 10.3389/fpsyg.2019.01735 31417462 PMC6685004

[28] Bastien CH, Vallières A, Morin CM. Validation of the insomnia severity index as an outcome measure for insomnia research. Sleep Med. 2001;2: 297–307. doi: 10.1016/s1389-9457(00)00065-4 11438246

[29] Alföldi P, Wiklund T, Gerdle B. Comorbid insomnia in patients with chronic pain: A study based on the Swedish quality registry for pain rehabilitation (SQRP). Disabil Rehabil. 2014;36: 1661–1669. doi: 10.3109/09638288.2013.864712 24320022

[30] TANG NKY, WRIGHT KJ, SALKOVSKIS PM. Prevalence and correlates of clinical insomnia co-occurring with chronic back pain. J Sleep Res. 2007;16: 85–95. doi: 10.1111/j.1365-2869.2007.00571.x 17309767

[31] Edinger J, Sanchez Ortuño M, Stechuchak K, Coffman C, Krystal A. Can CBT for insomnia also improve pain sensitivity in fibromyalgia patients?: results from a randomized clinical trial. Sleep Med. 2013;14: e213. doi: 10.1016/j.sleep.2013.11.509

[32] Aloush V, Gurfinkel A, Shachar N, Ablin JN, Elkana O. Physical and mental impact of COVID-19 outbreak on fibromyalgia patients. Clin Exp Rheumatol. 2021;39: S108–S114. doi: 10.55563/clinexprheumatol/rxk6s4 33734970

[33] Gammoh OS, Al-Smadi A, Tayfur M, Al-Omari M, Al-Katib W, Zein S, et al. Syrian female war refugees: preliminary fibromyalgia and insomnia screening and treatment trends. Int J Psychiatry Clin Pract. 2020;24: 387–391. doi: 10.1080/13651501.2020.1776329 32657625

[34] Guggisberg AG, Dalal SS, Zumer JM, Wong DD, Dubovik S, Michel CM, et al. Localization of cortico-peripheral coherence with electroencephalography. Neuroimage. 2011;57: 1348–1357. doi: 10.1016/j.neuroimage.2011.05.076 21672634

[35] Dalal SS, Zumer JM, Guggisberg AG, Trumpis M, Wong DDE, Sekihara K, et al. MEG/EEG source reconstruction, statistical evaluation, and visualization with NUTMEG. Comput Intell Neurosci. 2011;2011: 17. doi: 10.1155/2011/758973 21437174 PMC3061455

[36] Stenroos M, Mäntynen V, Nenonen J. A Matlab library for solving quasi-static volume conduction problems using the boundary element method. Comput Methods Programs Biomed. 2007;88: 256–263. doi: 10.1016/j.cmpb.2007.09.004 18022274

[37] Sekihara K, Nagarajan SS, Poeppel D, Marantz A. Asymptotic SNR of scalar and vector minimum-variance beanformers for neuromagnetic source reconstruction. IEEE Trans Biomed Eng. 2004;51: 1726–1734. doi: 10.1109/TBME.2004.827926 15490820 PMC4041989

[38] Sekihara K, Nagarajan SS, Poeppel D, Marantz A. Asymptotic SNR of scalar and vector minimum-variance beanformers for neuromagnetic source reconstruction. IEEE Trans Biomed Eng. 2004;51: 1726–1734. doi: 10.1109/TBME.2004.827926 15490820 PMC4041989

[39] Pernet C, Garrido MI, Gramfort A, Maurits N, Michel CM, Pang E, et al. Issues and recommendations from the OHBM COBIDAS MEEG committee for reproducible EEG and MEG research. Nat Neurosci. 2020;23: 1473–1483. doi: 10.1038/s41593-020-00709-0 32958924

[40] Haenschel C, Baldeweg T, Croft RJ, Whittington M, Gruzelier J. Gamma and beta frequency oscillations in response to novel auditory stimuli: A comparison of human electroencephalogram (EEG) data with in vitro models. Proc Natl Acad Sci U S A. 2000;97: 7645–7650. doi: 10.1073/pnas.120162397 10852953 PMC16599

[41] von Rotz R, Kometer M, Dornbierer D, Gertsch J, Salomé Gachet M, Vollenweider FX, et al. Neuronal oscillations and synchronicity associated with gamma-hydroxybutyrate during resting-state in healthy male volunteers. Psychopharmacology (Berl). 2017;234: 1957–1968. doi: 10.1007/s00213-017-4603-z 28429067

[42] Guggisberg AG, Rizk S, Ptak R, Di Pietro M, Saj A, Lazeyras F, et al. Two Intrinsic Coupling Types for Resting-State Integration in the Human Brain. Brain Topogr. 2015;28: 318–329. doi: 10.1007/s10548-014-0394-2 25182143

[43] Newman MEJ. Analysis of weighted networks. Phys Rev E—Stat Physics, Plasmas, Fluids, Relat Interdiscip Top. 2004;70: 9. doi: 10.1103/PhysRevE.70.056131 15600716

[44] Dubovik S, Pignat JM, Ptak R, Aboulafia T, Allet L, Gillabert N, et al. The behavioral significance of coherent resting-state oscillations after stroke. Neuroimage. 2012;61: 249–257. doi: 10.1016/j.neuroimage.2012.03.024 22440653

[45] Mottaz A, Solcà M, Magnin C, Corbet T, Schnider A, Guggisberg AG. Neurofeedback training of alpha-band coherence enhances motor performance. Clin Neurophysiol. 2015;126: 1754–1760. doi: 10.1016/j.clinph.2014.11.023 25540133

[46] Singh KD, Barnes GR, Hillebrand A. Group imaging of task-related changes in cortical synchronisation using nonparametric permutation testing. Neuroimage. 2003;19: 1589–1601. doi: 10.1016/s1053-8119(03)00249-0 12948714

[47] Amunts K, Mohlberg H, Bludau S, Zilles K. Julich-Brain: A 3D probabilistic atlas of the human brain’s cytoarchitecture. Science (80-). 2020;369: 988–992. doi: 10.1126/science.abb4588 32732281

[48] Neugebauer V, Li W, Bird GC, Han JS. The amygdala and persistent pain. Neuroscientist. Neuroscientist; 2004. pp. 221–234. doi: 10.1177/1073858403261077 15155061

[49] Thompson JM, Neugebauer V. Amygdala Plasticity and Pain. Pain Research and Management. Hindawi Limited; 2017. doi: 10.1155/2017/8296501 PMC5742506 29302197

[50] Foerster BR, Petrou M, Edden RAE, Sundgren PC, Schmidt-Wilcke T, Lowe SE, et al. Reduced insular γ-aminobutyric acid in fibromyalgia. Arthritis Rheum. 2012;64: 579–583. doi: 10.1002/art.33339 21913179 PMC3374930

[51] Spampanato J, Polepalli J, Sah P. Interneurons in the basolateral amygdala. Neuropharmacology. Elsevier Ltd; 2011. pp. 765–773. doi: 10.1016/j.neuropharm.2010.11.006 21093462

[52] Seeber M, Cantonas LM, Hoevels M, Sesia T, Visser-Vandewalle V, Michel CM. Subcortical electrophysiological activity is detectable with high-density EEG source imaging. Nat Commun. 2019;10: 1–7. doi: 10.1038/s41467-019-08725-w 30765707 PMC6376013

[53] Nahum L, Gabriel D, Spinelli L, Momjian S, Seeck M, Michel CM, et al. Rapid consolidation and the human hippocampus: Intracranial recordings confirm surface EEG. Hippocampus. 2011;21: 689–693. doi: 10.1002/hipo.20819 20865742

[54] Michel CM, Koenig T, Brandeis D, Gianotti LRR, Wackermann J. Electrical neuroimaging. Electrical Neuroimaging. Cambridge University Press; 2009. doi: 10.1017/CBO9780511596889

[55] Michel CM, Brunet D. EEG source imaging: A practical review of the analysis steps. Front Neurol. 2019;10: 325. doi: 10.3389/fneur.2019.00325 31019487 PMC6458265

[56] Sharma P, Scherg M, Pinborg LH, Fabricius M, Rubboli G, Pedersen B, et al. Ictal and interictal electric source imaging in pre-surgical evaluation: a prospective study. Eur J Neurol. 2018;25: 1154–1160. doi: 10.1111/ene.13676 29751364

[57] De Stefano P, Carboni M, Pugin D, Seeck M, Vulliémoz S. Brain networks involved in generalized periodic discharges (GPD) in post-anoxic-ischemic encephalopathy. Resuscitation. 2020;155: 143–151. doi: 10.1016/j.resuscitation.2020.07.030 32795598

[58] Lopes da Silva FH. Intracerebral Sources Reconstructed on the Basis of High-Resolution Scalp EEG and MEG. Brain Topography. Springer New York LLC; 2019. pp. 523–526. doi: 10.1007/s10548-019-00717-9 31129753

[59] Hsiao FJ, Wang SJ, Lin YY, Fuh JL, Ko YC, Wang PN, et al. Altered insula–default mode network connectivity in fibromyalgia: a resting-state magnetoencephalographic study. J Headache Pain. 2017;18. doi: 10.1186/s10194-017-0799-x 28831711 PMC5567574

[60] Yuan P, Raz N. Prefrontal cortex and executive functions in healthy adults: A meta-analysis of structural neuroimaging studies. Neuroscience and Biobehavioral Reviews. Elsevier Ltd; 2014. pp. 180–192. doi: 10.1016/j.neubiorev.2014.02.005 PMC4011981 24568942

[61] Ong WY, Stohler CS, Herr DR. Role of the Prefrontal Cortex in Pain Processing. Molecular Neurobiology. Humana Press Inc.; 2019. pp. 1137–1166. doi: 10.1007/s12035-018-1130-9 PMC6400876 29876878

[62] Tanwar S, Mattoo B, Kumar U, Bhatia R. Repetitive transcranial magnetic stimulation of the prefrontal cortex for fibromyalgia syndrome: a randomised controlled trial with 6-months follow up. doi: 10.1186/s42358-020-00135-7 32600394

[63] Folloni D, Sallet J, Khrapitchev AA, Sibson N, Verhagen L, Mars RB. Dichotomous organization of amygdala/temporal-prefrontal bundles in both humans and monkeys. Elife. 2019;8. doi: 10.7554/eLife.47175 31689177 PMC6831033

[64] Pomares FB, Roy S, Funck T, Feier NA, Thiel A, Fitzcharles MA, et al. Upregulation of cortical GABAA receptor concentration in fibromyalgia. Pain. 2020;161: 74–82. doi: 10.1097/j.pain.0000000000001707 31569142 PMC6940028

[65] Christian EP, Snyder DH, Song W, Gurley DA, Smolka J, Maier DL, et al. EEG-β/γ spectral power elevation in rat: a translatable biomarker elicited by GABA _Aα2/3_ -positive allosteric modulators at nonsedating anxiolytic doses. J Neurophysiol. 2015;113: 116–131. doi: 10.1152/jn.00539.2013 25253471

[66] Baumgarten TJ, Oeltzschner G, Hoogenboom N, Wittsack H-J, Schnitzler A, Lange J. Beta Peak Frequencies at Rest Correlate with Endogenous GABA+/Cr Concentrations in Sensorimotor Cortex Areas. Johnson B, editor. PLoS One. 2016;11: e0156829. doi: 10.1371/journal.pone.0156829 27258089 PMC4892568

[67] Gaetz W, Edgar JC, Wang T PL Roberts DJ, Gaetz W. Relating MEG Measured Motor Cortical Oscillations to resting γ-Aminobutyric acid (GABA) Concentration. Neuroimage. 2011;55: 616–621. doi: 10.1016/j.neuroimage.2010.12.077 21215806 PMC3411117

[68] Gao Y, Wang Q, Ding Y, Wang C, Li H, Wu X. Selective Attention Enhances Beta-Band Cortical Oscillation to Speech under “Cocktail-Party” Listening Conditions. 2017;11: 1–10. doi: 10.3389/fnhum.2017.00034 28239344 PMC5300994

[69] Piai V, Roelofs A, Rommers J, Maris E. Beta oscillations reflect memory and motor aspects of spoken word production. Hum Brain Mapp. 2015;36: 2767–2780. doi: 10.1002/hbm.22806 25872756 PMC6869587

